# Applications of Melanin and Melanin-Like Nanoparticles in Cancer Therapy: A Review of Recent Advances

**DOI:** 10.3390/cancers13061463

**Published:** 2021-03-23

**Authors:** Stefania Cuzzubbo, Antoine F. Carpentier

**Affiliations:** 1 Université de Paris, PARCC, INSERM U970, 75015 Paris, France; stefania.cuzzubbo@inserm.fr; 2 Laboratoire de Recherches Biochirurgicales (Fondation Carpentier), Assistance Publique-Hôpitaux de Paris (AP-HP), Hôpital Européen Georges Pompidou, 75015 Paris, France; 3 Université de Paris, Paris Diderot, 75010 Paris, France; 4 Assistance Publique-Hôpitaux de Paris (AP-HP), Hôpital Saint-Louis, Service de Neurologie, 1, Avenue Claude Vellefaux, 75010 Paris, France

**Keywords:** melanin, cancer vaccine, adjuvant, nanoparticles, L-DOPA, dopamine, immunotherapy, photothermal therapy, vaccine, lymphocytes, CD8

## Abstract

**Simple Summary:**

Therapeutic vaccines represent an attractive strategy for cancer patients but have not yet achieved significant efficacy. Many strategies are currently being explored to improve their efficacy and, notably, different types of adjuvants are under development. Nanoparticle systems are promising adjuvants as they act as carriers for antigens to target antigen-presenting cells in lymph nodes. Among them, melanin-based nanoparticles are particularly interesting since they efficiently carry antigens into draining lymphoid tissues and display immunomodulatory properties. In addition, melanin-based nanoparticles can also play an active role in triggering anti-cancer responses in the context of photothermal therapy. This review summarizes the promising results of the melanin-based cancer vaccines recently reported in preclinical models.

**Abstract:**

Thanks to the growing knowledge about cancers and their interactions with the immune system, a huge number of therapeutic cancer vaccines have been developed in the past two decades. Despite encouraging results in pre-clinical models, cancer vaccines have not yet achieved significant clinical efficacy. Several factors may contribute to such poor results, including the difficulty of triggering a strong immune response and the immunosuppressive tumor microenvironment. Many strategies are currently being explored. Different types of adjuvants have been incorporated into vaccine formulations to improve their efficacy, as cancer antigens are usually poorly immunogenic. Nanoparticle systems are promising tools as they act as carriers for antigens and can be surface-modified so that they specifically target antigen-presenting cells in lymph nodes. Bioinspired nanomaterials are ideal candidates thanks to their biocompatibility. Recently, melanin-based nanoparticles were reported to efficiently localize into draining lymphoid tissues and trigger immune responses against loaded antigens. In addition, by virtue of their photochemical properties, melanin-based nanoparticles can also play an immunomodulatory role to promote anti-cancer responses in the context of photothermal therapy. In this review, we discuss the above-mentioned properties of melanin, and summarize the promising results of the melanin-based cancer vaccines recently reported in preclinical models.

## 1. Introduction

Cancer is one of the world’s leading health problems. It is estimated that every sixth death in the world is due to cancer (global burden of disease) [1]. Many efforts have been made to improve cancer prognosis, and the recent successes of immune checkpoint inhibitors have certainly increased hopes. However, these molecules have proven their efficacy only in a limited subset of patients. Therapeutic vaccines represent an attractive strategy for cancer patients, but have not yet achieved significant clinical efficacy. To date, only one vaccine is approved: Provenge^®^, which is indicated for prostate cancer patients [2]. Anti-tumor efficacy has been demonstrated for a large number of vaccine formulations in numerous murine models as well as in early phase trials, but their efficacy has not yet been confirmed in phase III trials. Difficulties in triggering a strong tumor-specific immune response, in addition to the immunosuppressive tumor microenvironment, are probably the major reasons for such a lack of efficacy. In that respect, the optimization of adjuvants in vaccine formulations plays a key role in boosting the immune response through several mechanisms, primarily: (1) by carrying the antigen into the lymph nodes and prolonging its availability in that site; (2) by activating the innate immunity; and (3) through reversing the tumor-associated immune suppression [3].

**Figure 1:**
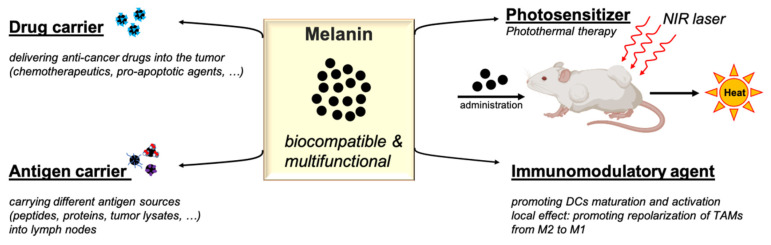
Schematic illustration of the applications of melanin-based platforms in cancer therapy. NIR: Near-infrared; TAM: tumor-associated macrophage.

Nanoparticle systems have attracted much attention in the field of cancer vaccines as they can operate as carriers for different antigens to the lymph nodes, thus specifically targeting antigen-presenting cells. In addition, nanoparticles also display some immunostimulatory properties. Bioinspired nanomaterials are of special interest in that field because of their biocompatibility. Melanin-based nanoparticles efficiently localize into draining lymphoid tissues and exhibit immunostimulatory abilities in different cancer vaccine formulations. In addition, the unique photophysical and photochemical properties of melanin enable its use as a photosensitizer in photothermal therapy (Figure 1). However, the term “melanin” includes a multitude of substances (natural and synthetic) with different biochemical structures which might not display similar immune properties. In this review, we discuss the above-mentioned properties of the different melanins, and summarize the promising results of the melanin-based cancer vaccines recently reported in preclinical models.

## 2. Synthesis of Melanin in Mammals

**Figure 2:**
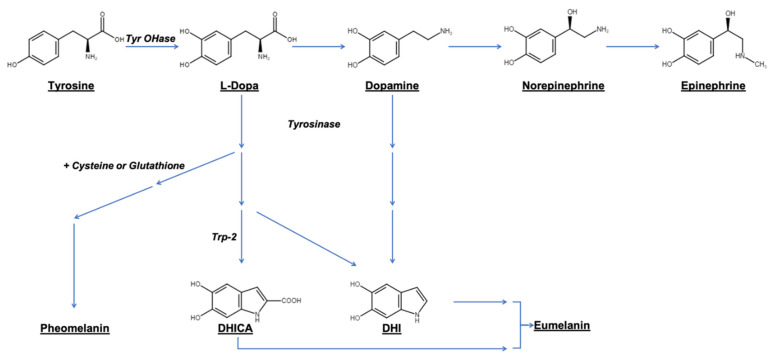
Schematic illustration of main metabolic pathways for eumelanin and pheomelanin synthesis. DHICA: dihydroxyindole carboxylic acid; DHI: 5, 6-dihydroxyindole.

Tyrosine plays a key role in mammalian life and is involved in major biological pathways such as catecholamine synthesis and melanogenesis. By the action of tyrosine hydroxylase, L-tyrosine is first hydroxylated to obtain L-3,4-dihydroxyphenylalanine (L-DOPA). L-DOPA can then be decarboxylated to form dopamine, noradrenaline, and adrenaline, which act as neurotransmitters [4]. Alternatively, L-DOPA and dopamine have the ability to undergo oxidative polymerization, generating dark polymer pigments named melanin via a series of enzymatic and non-enzymatic reactions (Figure 2) [5]. In mammals, two main types of melanin have been described: (1) eumelanins, produced by serial oxidations of L-DOPA and dopamine and leading mainly to copolymers of dihydroxyindole carboxylic acid (DHICA) and 5, 6-dihydroxyindole (DHI), although the exact chemical structure has not yet been elucidated; and (2) pheomelanins, in which L-DOPA undergoes cysteinylation by conjugation with glutathione or cysteine before its oxidative polymerization [6]. In addition to eumelanin and pheomelanin, neuromelanin is produced in specific populations of catecholaminergic neurons in the brain, and results from the oxidative polymerization of epinephrine and norepinephrine. In non-mammalian species such as insects, fungi, and plants, the oxidation or polymerization of various compounds lead to different melanin types named allomelanins [7]. “Melanin” is thus a broad and generic term for designating a group of natural pigments found in most organisms and is usually produced by the oxidative polymerization of chemical compounds containing a catechol ring.

In mammals, melanin is mainly produced by melanocytes found in the basal layer of the epidermis and is responsible for hair and skin pigmentation. Pigmentation, which protects tissues against mutagenic light, is the most important and well-recognized function of melanin. Interestingly, melanin can also be found outside the skin in mammals, such as in the iris of the eye, the stria vascularis of the inner ear, and sometimes in visceral organs, but their physiological role remains unclear [5]. Several functions for this extra-dermal melanin have been suggested, including protection against oxidative stress (free radical scavenger) or metal homeostasis (through functional groups with affinity for metal ions binding) [8]. In addition, the waves of pigmentation of murine spleens (in a phenomenon that is denominated as splenic melanosis) have been known of for decades [9], but the physiological role of this intriguing process remains unclear. Similarly, in lower vertebrates such as fish, the presence of melanin can be found in various organs within melanomacrophages, with these histiocytic cells probably being responsible for the removal of foreign pathogens [10].

## 3. Synthesis of Melanin-Like Nanoparticles

The unique characteristics of melanin and its potential applications in medicine have encouraged scientists to develop melanin nanoparticles. Melanin-based nanoparticles can be obtained by biological extraction or chemical synthesis [11]. The former method consists in physically separating and purifying melanin nanoparticles from different natural sources (cuttlefish ink, black sesame, hair) and processing these materials to obtain water-soluble melanin nanoparticles. These procedures depend upon the starting material, cannot be standardized, and the resulting extracts have unclear chemical structure and contaminants. For instance, Deng et al. reported that nanoparticles extracted from cuttlefish ink contained amino acids and monosaccharides besides melanin [12]. Compared to natural melanin, synthetic melanin-like nanoparticles offer the advantage of standardized physicochemical procedures as well as better reproducibility. Indeed, by controlling synthesis processes, melanin nanoparticles of different sizes, morphologies, and molecular structures can be synthetized. There are three main methods to synthetize nanoparticles from dopamine (or sometimes L-DOPA). The most commonly used method is using chemical oxidation by adding an oxidant at a basic pH. Briefly, in this method L-DOPA or dopamine hydrochloride are added to alkaline solution (pH > 8) under stirring. Dopamine or L-DOPA monomers hence undergo oxidation reaction under oxygen action and polymerize into melanin. The size of melanin nanoparticles can be easily modified depending on the concentrations of chemical products and pH [11]. The other two methods of melanin synthesis are more difficult to perform. The enzymatic method replicates the natural synthesis of melanin through the use of tyrosinase or laccase enzymes, but this process requires functional enzymes that should be eventually purified. The third method consists of promoting the polymerization of dopamine by electrolysis. This method allows for the direct deposit of melanin nanoparticles on the surface of materials (if the latter is conductive) but the resulting product should be considered more as a dopamine-coated material than true melanin nanoparticles [13].

## 4. Melanin and Its Putative Role in Immunity

Several facts suggest that immunity and pigmentation could be functionally linked.

(a) Alpha melanocyte-stimulating hormone (α-MSH), an endogenous peptide hormone of the melanocortin family, plays a key role in melanogenesis through binding to its receptor MC1R (melanocortin 1 receptor). It appears that α-MSH has a wide range of activities that include anti-inflammatory effects and immunomodulation on macrophages and neutrophils [14]. These anti-inflammatory effects have been well established in several models of inflammatory or ischemia/reperfusion injury and bacterial endotoxin-induced inflammation [15]. In addition, α-MSH can promote regulatory T-lymphocytes (Treg), which modulate immunity-targeting specific antigens [16].
(b) Melanogenesis plays a role in invertebrate innate immunity. Insects commonly activate the formation of melanin around intruding microorganisms in a process known as melanization [17]. Within minutes after infection, microbes are encapsulated within melanin and the generation of free radical byproducts during the formation of this capsule is thought to aid in killing them. This process constitutes a major aspect of the innate immune defense system against invading pathogens in invertebrates but does not occur in mammals. The situation might even be the opposite in mammals, as melanin synthesis by *Cryptococcus neoformans* actually increases its virulence by protecting the fungus against phagocytic killing by the host [18]. Moreover, different strains of mice that differed only in the gene encoding tyrosinase, a key enzyme in the synthesis of melanin, showed no difference in the clinical course of malaria infection [19]. Finally, synthetic melanin suppresses cytokine production in macrophages stimulated with lipopolysaccharide [20].
(c) The dendritic nature of melanocytes and their strategic location in the skin raised the idea that they could play a role in adaptive immunity to external pathogens [21]. Melanocytes indeed exhibit phagocytic functions, and phagosomes are transported from the cell surface to the melanosomes that contain many lysosomal enzymes [22,23]. Further studies have shown that melanocytes can act as antigen-presenting cells [22,24]. In addition, in mice with melanocytosis, melanin granules in the skin are continuously captured and transported to regional lymph nodes by Langerhans cells [25,26]. However, in the literature it has not been mentioned that naturally occurring melanin can promote an adaptive immune response such as through antibodies or cytotoxic T-lymphocytes in vivo.

## 5. Potential Applications of Melanin in Medicine

**Figure 3:**
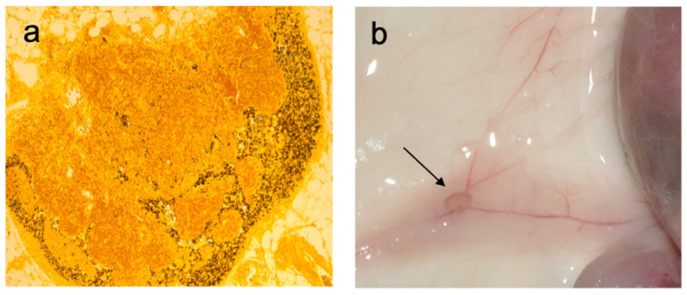
Melanin deposits within lymph nodes in vivo in Balb/c mice mice, 48 h after subcutaneous injection of 100 µg of synthetic L-3,4-dihydroxyphenylalanine (L-DOPA) melanin. (**a**) Fontana–Masson staining of a draining lymph node, showing melanin-laden macrophages in the sinuses and in the paracortical area; (**b**) macroscopic aspect of the draining inguinal lymph nodes (arrow) (data from our lab).

Several types of nanoparticles are under development for use in drug delivery systems. Table 1 summarizes the characteristics of the main nanoparticles under development in cancer therapy strategies. Among them, melanin has the important advantage of already being present in the human body as nanosized particles in melanocytes. Hence, the native biocompatibility of melanin nanoparticles makes it a safe biopolymer for new applications in the biomedical field. Indeed, melanin pigments display unique physicochemical properties that can be advantageously used for the clinical development of drug delivery systems, imaging, or theranostics. The most interesting physicochemical characteristics reside in their ability to bind molecules and absorb light. Given its ability to bind molecules, melanin can be used as a drug carrier [27,28]. Indeed, the various π-conjugated structures of melanin can bind with various aromatic structures via π–π stacking. As an alternative, drugs can be linked through covalent bonds to the melanin surface through the presence of functional groups such as catechol, o-quinone, amine, and imine, or also physically encapsulated within the polymer matrix (non-covalent loading) [29]. Furthermore, in vitro synthesis of melanin offers the possibility to manipulate the size and surface characteristics of the particles. Consequently, both the loading efficiency and the release of the drug can be controlled, as the drug adsorption capability of synthetic melanin is dependent on its surface area [30]. Melanin, once injected, makes a local depot for several weeks, allowing local release. Melanin is also partially driven to the draining lymph nodes (Figure 3) and can thus be used as a carrier for a molecule to reach the lymphatic system [31]. Most interestingly, melanin migration into the lymph nodes is also encountered in humans. Paracortical melanin deposits within the lymph nodes, closely mimicking what is observed in mice, are observed in the case of skin disease in a phenomenon known as dermatopathic lymphadenopathy [32]. These biodistribution properties towards the lymph nodes can be particularly useful in vaccine approaches, as detailed below. Alternatively, active targeted delivery can be obtained by attaching specific ligands to the melanin nanoparticle surface [33,34,35]. Drug-loaded melanin-like nanoparticles can efficiently target cell organelles and, notably, mitochondria [36].

As mentioned above, melanin is an efficient photosensitizer, being able to absorb light energy and convert it into heat. Selective heating of melanin within tissues can be thus achieved and used in photothermal therapy (PTT). PTT has emerged as a minimally invasive approach to kill specific cells without damaging the surrounding healthy tissue. This strategy is particularly relevant with melanin, given its large absorption coefficient in the near infrared region. This range of wavelengths is useful for therapeutic applications as tissues show minimum absorption in this region in the spectrum, thus enhancing penetration and targeting efficiency [50]. Melanin nanoparticles synthetized from dopamine or from associated arginine and dopamine have demonstrated a strong conversion efficiency [51,52]. Synthetic melanin nanoparticles possess an additional advantage in that they can be readily surface-modified, thus allowing the enrichment of photothermal conversion agents. Moreover, compared to other materials, melanin is a biocompatible and non-toxic agent. Other organic agents have been approved by the US Food and Drug Administration (FDA) for clinical imaging, such as indocyanine green (ICG). However, severe photobleaching and instability limit the further applications of these molecule dyes in PTT [11]. Several inorganic nanomaterials certainly hold excellent photothermal properties but given their high cost and/or poor biodegradability, researchers are more attracted by strategies developing safe photosensitizer agents from natural materials, such as melanin. In order to further increase the biocompatibility of melanin, Jiang et al. coated natural melanin nanoparticles extracted from cuttlefish with red blood cell membranes. This strategy favored the accumulation of melanin at the tumor site following intravenous injection [53].

Moreover, multifunctional melanin-like nanoparticles have been designed that combine tumor targeting, photothermal properties, and local drug release [54,55]. Melanin-based photothermal therapy is of particular interest in the field of cancer immunotherapy as it can directly kill tumor cells, hence promoting tumor antigen release and ultimately immune-mediated anti-tumor responses. Potential applications of melanin in cancer immunotherapy will be described below.

### 5.1. Melanin as an Adjuvant in Cancer Vaccines

**Figure 4:**
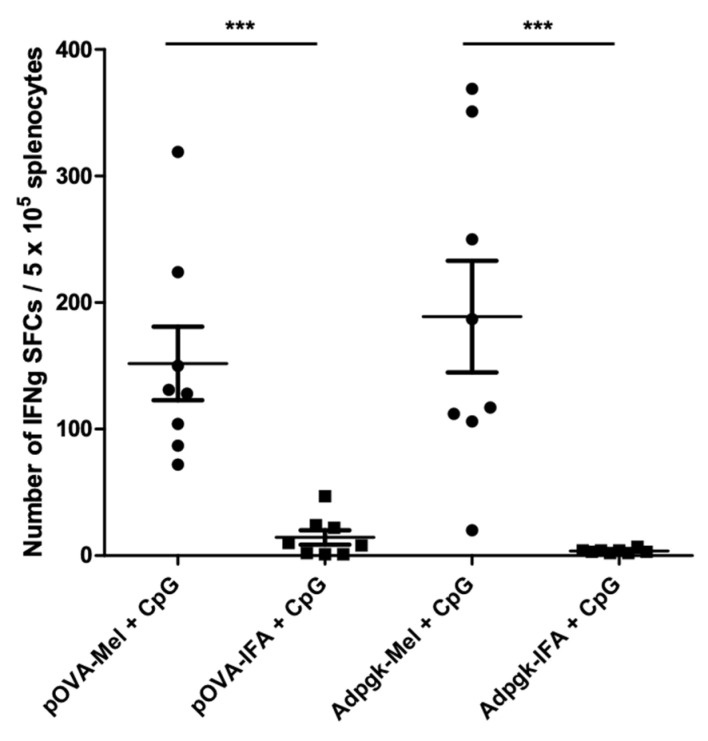
A vaccine formulation based on L-DOPA melanin triggers robust T cell responses against short and long peptides. ELISpot analysis of splenocytes on day 21 after subcutaneous immunizations on days 0 and 14 of C57/Bl6 mice with the indicated formulation containing the long peptide pOVA (SMLVLLPKKVSGLKQLESIINFEKLTKWTS) or the short peptide mAdpgk (KKASMTNMELM). CpG: 5′-TAAACGTTATAACGTTATGACGTCAT (Oligovax, Paris, France); IFA: incomplete Freund’s adjuvant (Sigma-Aldrich, Saint-Quentin-Fallavier, France). IFNg: interferon gamma; SFC: spot-forming cell. Each point represents an individual mouse (*n* = 8 mice/group with pooled data from 2 different experiments). Bars: mean values ± standard error of the mean (SEM). *** *p* < 0.001. For more detail on methods, see Cuzzubbo et al. 2020 [57].

**Figure 5:**
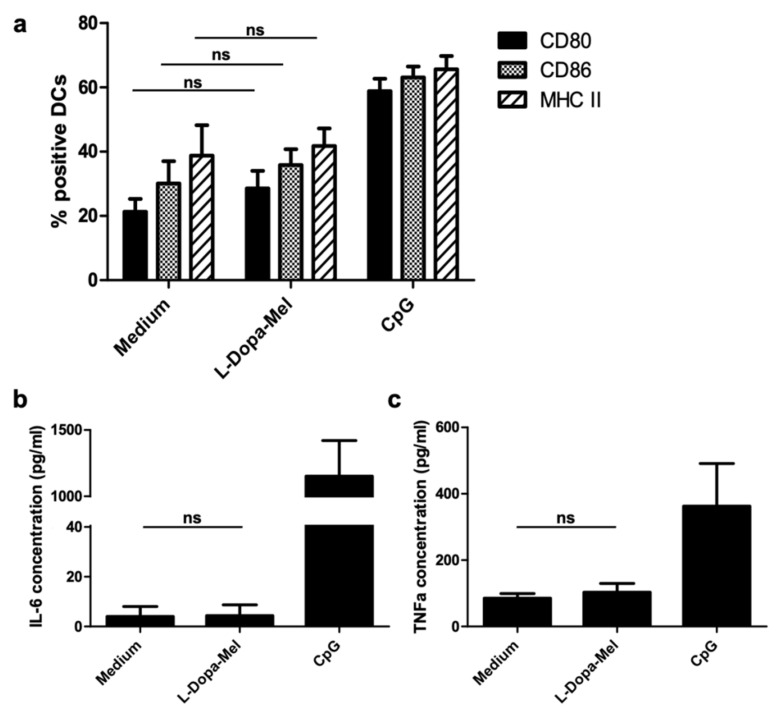
Effects of L-DOPA-melanin on dendritic cells. Cells derived from the bone marrow of C47-Bl6 mice were cultivated with GM-CSF (granulocyte-macrophage colony-stimulating factor) at 20 ng/mL for 8 days and then non-adhering cells incubated with L-DOPA melanin at 4 µg/mL or CpG at 1 µg/mL (positive control; 5′-TAAACGTTATAACGTTATGACGTCAT, Oligovax, Paris, France) or medium alone (negative control) for 24 h (5 × 10^5^ cells/mL). Cells were then analyzed by flow cytometry (**a**) and supernatant by ELISA (**b**,**c**). (**a**) After incubation with anti-mouse CD16/32 (clone 93, Biolegend) and Amcyan LIVE/DEAD Fixable Aqua Dead Cell Stain Kit (Invitrogen) at room temperature for 15 min, cells were stained with anti-mouse CD11c PC (clone N418, Biolegend), CD80 FITC (clone 16–10A1, eBioscience), CD86 PE-Cy7 (clone GL−1, Biolegend), and major histocompatibility complex class II (MHC II) PE (clone NIMR−4, eBioscience). The graph shows the percentage of CD80-, CD86- and MHC II-positive cells among CD11c+ cells. (**b**,**c**) The amount of proinflammatory cytokines (B: interleukin 6, IL-6; C: tumor necrosis factor alpha, TNF-a) in the cell culture supernatants was determined by ELISA. Data from 3 independent experiments with triplicates as mean ± SEM; statistical analysis was performed with Mann–Whitney test; ns: non-significant (*p* > 0.05) (data from our lab). APC: antigen-presenting cell; DC: dendritic cell.

Melanin is an excellent candidate for drug delivery system given its biocompatibility, biodegradability, high loading efficiency, and the possibility of manipulating both binding and release of drugs [45]. Due to such unique features, melanin is under development as a carrier for antigenic peptides in vaccines. Peptides are widely used as an antigen source because of their low toxicity, low cost, and the presence of direct functional T-cell epitopes [45]. However, free peptides rapidly reach the systemic circulation after subcutaneous injection and only a small portion of the injected dose is captured by antigen-presenting cells (APCs). Effective antigen presentation by APC to T-cells is a prerequisite for triggering an anti-tumor immune response. Cytotoxic T lymphocytes are efficiently activated only if antigens are presented together with MHC (major histocompatibility complex) class I molecules, along with the simultaneous expression of costimulatory molecules by APCs. Nanoparticulated formulations have attracted a lot of interest as some of them are able to not only improve the antigen presentation but also to trigger inflammatory responses in dendritic cells [3]. The size of nanoparticles seems to be a determinant for migration towards lymph nodes. Diameters between 20 and 200 nm have been reported to more likely enter lymphatic capillaries, reach lymph nodes, and finally be phagocyted by dendritic cells and macrophages [56]. Several nanoparticulated carriers of this size are under development for peptide vaccines and, among these, melanin-like particles have the important advantage of being nontoxic and biomimetic. Other nanoparticles, such as carbon nanotubes and cationic polymers, have proven their efficacy in both delivering antigens and triggering immune response, but the toxicity issue is still a concern for some of them. Our group has performed pioneering studies using a vaccine formulation based on a synthetic L-DOPA melanin loaded with synthetic peptides. After sub-cutaneous injection in mice, melanin–peptide complexes reach the sinusal and paracortical zones of the draining lymph nodes. These areas are T-cell zones and this spontaneous targeting could thus explain the very efficient presentation of antigens to T-cells (both CD4 and CD8 + T-lymphocytes) [31]. Our vaccine formulation showed encouraging results in mice, triggering robust CD8+ T-lymphocyte responses against several antigens (short and long peptides) even with very low doses of antigen, especially—but not necessarily—when combined with the immunostimulatory adjuvant Toll like receptor (TLR9) agonist (CpG) (Figure 4). This formulation was more efficient than the classic combination of incomplete Freund adjuvant (IFA) and CpG in terms of both immune response (amount of circulating and intratumor specific CD8 T cells) and anti-tumor efficacy (inhibition of tumor growth) [57]. L-DOPA melanin seems to act mainly as a carrier for antigen, with limited immunostimulant activity, as the culture of bone marrow-derived dendritic cells (BMDCs) with L-DOPA melanin did not cause any significant activation of these cells (Figure 5).

Other melanin-like nanoparticles have been proposed as cancer vaccine strategies. Wang et al. reported a vaccine based on a dopamine nanoparticle carrier. Similar to L-DOPA melanin, the formulation allowed peptide to reach draining lymph nodes and prolonged its retention up to 48 h, compared to only 12 h for the free peptide. This vaccine formulation induced in vitro activation and maturation of bone marrow-derived dendritic cells and was also able to trigger an immune cytotoxic response along with a minor but significant effect on tumor growth in vivo [58]. In another work, polydopamine nanoparticles showed direct anti-cancer activity, meaning that they are capable of killing cancer cells in vitro at a concentration of 0.02–0.04 mg/mL, but not healthy fibroblasts [59].

Melanin possesses the interesting feature of being able to stimulate the immune system in certain conditions [12,46,60]. Indeed, attention should be paid to the different effects exhibited by melanin depending on its composition and its origin (natural versus synthetic melanin-like particles). Actually, most of the immune effects induced by melanin have been obtained with natural extracted melanin. El-Obeid´s group reported immunostimulatory effects on human monocytes and cytotoxic effects towards cancer cells with a plant melanin (*Nigella sativa* L.). For the latter activity, only high concentrations of herbal melanin (500 µg/mL) were actually capable of inducing apoptosis in THP−1 cells (human monocytic leukemia cell line) via the TLR4 pathway, thus limiting its benefit in clinical practice, as these concentrations are detrimental for healthy cells [46,47]. Deng et al. reported more promising results in the field of cancer therapy with melanin extracted from cuttlefish ink [12]. The authors used a non-purified melanin which contained other molecules such as various oligopeptides, mono- and polysaccharides, metals, etc. Therefore, these biological effects cannot be fully attributed to melanin. Cuttlefish ink nanoparticles were able to activate dendritic cells in vitro and to repolarize macrophages from an immunosuppressive (M2) profile towards a proinflammatory phenotype (M1). This effect is of particular interest in cancer therapy, as M2 tumor-associated macrophages (TAMs) are one of the main drivers of immunosuppression in the tumor microenvironment. As discussed in more detail below, Deng et al. leveraged the unique properties of melanin to combine the photothermal-induced death of tumor cells and repolarization of TAMs that promotes T-cell infiltration into the tumor. A pro-inflammatory role has also been demonstrated for human neuromelanin. In the context of Parkinson’s disease, neuromelanin was in fact reported to activate microglia by triggering NK-**κ**B (nuclear factor kappa-light-chain-enhancer of activated B cells) activation and the release of the proinflammatory cytokines TNF-a (tumor necrosis factor alpha) and IL-6 (interleukin 6) in in vivo and in vitro studies [61]. Oberlander et al. confirmed these immunostimulant effects of neuromelanin towards BMDCs [60]. However, the opposite activities of melanin are well recognized and documented in fungal infections, and notably in interactions between melanin-producing fungi and macrophages. Melanin-producing *Cryptococcus* and *Aspergillus* are in fact highly invasive and can suppress the immune system of the host more efficiently than strains not producing melanin. In vitro studies confirmed the immunosuppressive role of melanin towards bone marrow-derived macrophages when stimulated simultaneously with fungal components [62].

In summary, melanin-like nanoparticles have shown very promising results in vaccines given their dual function of drug carrier and immunostimulant agent. However, the type of melanin needs to be selected for each vaccine formulation, and further studies are required to define the best melanin to be used as an adjuvant in vaccines.

### 5.2. Melanin as a Photosensitizer in Cancer Photothermal Therapy

Photothermal therapy (PTT) is of particular interest in cancer therapy. The direct killing effect of hyperthermia on cancer cells is associated with the activation of the immune system through the so-called immunogenic cell death (ICD). PTT induces the release of tumor antigens and immunostimulant molecules such as heat shock proteins and damage-associated molecular patterns (DAMPs), thus attracting and activating several immune cells (macrophages, dendritic cells, T cells) into the tumor site. By this immunological activity, the immediate killing-effect on cancer cells is boosted, and PTT contributes to establishing an immunological memory preventing tumor recurrence [63].

Considering the efficiency of melanin-based nanoparticles for both immunotherapy and PTT, Chen et al. developed a platform with polydopamine-coated Al2O3 nanoparticles. This nano-complex was co-administered with CpG into B16F10 tumors of mice and irradiated with a NIR (Near-infrared) laser. The PTT was thus responsible for the rapid tumor cell necrosis, whereas the Al2O3 combined with CpG resulted in the elimination of residual tumor cells by triggering a systemic immune response [64]. Another group developed iron-chelated melanin-like nanoparticles capable of hyper-thermally killing tumor cells via PTT and synergistically repolarizing tumor-associated macrophages from an immunosuppressive (M2) towards a pro-inflammatory phenotype (M1) [65]. As mentioned above, a similar approach was adopted by Deng et al., but with natural cuttlefish ink nanoparticles containing melanin. Cuttlefish ink treatment exhibited excellent ability to kill tumor cells through PTT and repolarized M2 tumor-associated macrophages, thus promoting T cell infiltration within the tumor [12]. Li et al. also used melanin nanoparticles extracted from cuttlefish ink coated with 4T1 cancer cell membranes that, surprisingly, displayed good tumor targeting after intravenous injections, allowing an efficient PTT-induced immunogenic cell death. Additionally, the intra-peritoneal administration of IDO (indoleamine-pyrrole 2,3-dioxygenase) inhibitor led to a synergistic effect, resulting in a powerful and persistent anti-tumor response [48].

A new approach to PTT has been promoted by Ye et al. They developed a melanin-mediated immunotherapy patch in which PTT was used to increase the antigen uptake by dendritic cells. To elaborate, B16F10 tumor lysate containing synthetic melanin was loaded into polymeric microneedles in order to allow sustained release of the lysate into the skin. Under near-infrared excitation, melanin mediated the generation of heat, which further promotes tumor-antigen uptake by dendritic cells. As a consequence, this approach increased the infiltration of polarized T cells and local cytokine release, resulting in a stronger anti-tumor response and increased survival of mice. However, authors did not mention the process of synthesis of melanin and no chemical analysis of its structure/composition was reported [66].

Another strategy to increase the efficacy of PTT is to combine it with targeted anti-cancer drugs. The high temperature generated by PTT is able to kill cancer cells but, at the same time, is detrimental to nearby normal tissues and favors inflammation and tumor metastasis [67]. New strategies to increase the efficacy of killing cancer cells at lower temperatures are thus emerging and, among them, the combination of PTT with targeted anti-cancer agents has provided encouraging results, especially in melanin-based PTT. The unique properties of melanin allow it to act as a drug delivery agent and photosensitizer at the same time. The acidity of the tumor environment induces the drug release and therefore a tumor-targeted action of various anti-cancer molecules such as chemotherapeutics [33,36,44,54,55] or apoptosis/autophagy inducers (Beclin 1) [35]. Furthermore, some anticancer drugs have immunostimulatory properties, thus making it possible to use a combined chemotherapy–PTT–immunotherapy strategy. It was in this context that He and collaborators designed a novel nanoplatform (DOX/PDA@Au@BV/PTX) based on hierarchical drug release and cascade therapy including localized PTT, systemic therapy, and elicited immune responses. Specifically, the first step was carried out by polydopamine releasing doxorubicin into lysosomes thanks to the acid pH. Then, gold and polydopamine acted as photosensitizers for PTT and biomimetic vesicles released paclitaxel, which promoted the third step of chemotherapy and triggered immune responses [68]. In this complex system, polydopamine plays a crucial role since gold nanomaterials need to be at high—toxic—concentrations to achieve photothermal efficacy. Polydopamine thus increased both biosafety and efficacy of the nanoplatform.

## 6. Conclusions

The large physicochemical variety of properties of melanin along with its inherent biocompatibility have inspired scientists to design novel melanin-like nanoparticles for diverse fields ranging from nanomedicine to nanocosmetics [45]. Melanin applications in photothermal therapy (PTT) and delivery drug systems have sparked attention in the field of cancer therapy. In one promising approach, melanin nanoparticles can be loaded with cancer antigens and used as cancer vaccines. We and others have shown the outstanding potential of melanin in such vaccines to trigger robust CD8+ T-lymphocyte responses against associated antigens. When added to vaccine formulations, melanin indeed promotes the presentation of the antigen to APCs by acting as an antigen carrier towards draining lymph nodes. In another approach, intratumoral melanin nanoparticles can efficiently deliver locally toxic agents or be used in PTT to increase cell cytotoxicity and antigen release.

However, the term “melanin” includes a multitude of substances with different properties and effects. Most reports insufficiently define the final composition and chemical structure of the melanin used, and those using natural melanin are not always able to clarify the respective roles played by melanin itself and by other associated molecules. This lack of information is mostly related to the inherent difficulties in characterizing melanin nanoparticles. A better knowledge of physicochemical properties of the different melanins along with a better understanding of the mechanisms behind anti-cancer activity for each type of melanin is required to optimize the applications of melanin in cancer therapy. However, promising preclinical data have already shown that melanin will probably have several applications in nanomedicine and immunotherapy.

## 7. Patents

The AP/HP (Assistance Publique des Hopitaux de Paris) filed a provisional patent application regarding the vaccine formulation based on synthetic melanin from L-Dopa. A.F. Carpentier is listed as the inventor. A.F. Carpentier holds shares in Altevax Inc. and is a consultant for the BMS. The authors declare that there are no other conflicts of interest.

## Figures and Tables

**Table 1:** Characteristic and advantages of the principal nanoparticles under development in cancer therapy.

Nanoparticle	Drug Loading/Releasing Capability	Manufacture	Cost of Production	Biocompatibility	Major Advantages	References
Liposomes	High and versatile drug loading: DNA, mRNA, proteins, peptides, immunostimulating agents	Complex synthesis Unstable product	High	Good biocompatibility, but improvement of the biodistribution in vivo is needed	Versatility: targeted design with the desired size, charge, and distribution	[3,37,38,39]
Chitosan	Low loading ratio	Easy synthesis Unstable product	Low	Good	Biocompatibility	[40]
Polylactic-co-glycolic acid (PLGA)	Poor loading ratio (<10%) with high burst release Versatility: chemotherapeutics, tumor lysate, DNA, mRNA, proteins, peptides, antibodies, genes	Complex synthesis relative to other nanoparticles Stable product	High	Good	Biocompatibility and biodegradability Reproducibility Versatility	[40,41,42]
Gold	High loading ratio	Reproducible synthesis, stable product	High	Cytotoxicity has been reported depending on the size and charge of gold nanoparticles	Controllable size, shape, and surface charges Ability to combine photothermal therapy (PPT) with delivery system activity	[43]
Synthetic melanin	High loading rate Versatility: chemotherapeutics, tumor lysate, proteins, peptides	Reproducible synthesis, stable product	Low	Good	Biocompatibility Reproducibility Versatility due to controllable size, shape, and surface charges Applications in cancer vaccine Ability to combine PPT with delivery system activity	[11,27,30,31,44]
Natural melanin	High loading rate	Poorly defined compounds, obtained by extraction from different sources	Low	Good	Biocompatibility Ability to combine PPT with delivery system activity Some evidence of direct immunostimulating effects	[12,45,46,47,48,49]

## Data Availability

All data cited in this review are showed in the manuscript.

## References

[1] GBD 2016 Causes of Death Collaborators (2017). Global, regional, and national age-sex specific mortality for 264 causes of death, 1980–2016: A systematic analysis for the Global Burden of Disease Study 2016. Lancet.

[2] Cheever M.A., Higano C.S. (2011). PROVENGE (Sipuleucel-T) in prostate cancer: The first FDA-approved therapeutic cancer vaccine. Clin. Cancer Res..

[3] Cuzzubbo S., Mangsbo S., Habra K., Nagarajan D., Pockley A.G., McArdle S.E. (2021). Cancer vaccines: Adjuvant potency, importance of age, lifestyle and treatments. Front. Immunol..

[4] Eisenhofer G., Tian H., Holmes C., Matsunaga J., Roffler-Tarlov S., Hearing V.J. (2003). Tyrosinase: A developmentally specific major determinant of peripheral dopamine. FASEB J..

[5] Slominski A., Tobin D.J., Shibahara S., Wortsman J. (2004). Melanin pigmentation in mammalian skin and its hormonal regulation. Physiol. Rev..

[6] Palumbo A., d’Ischia M., Misuraca G., Prota G. (1991). Mechanism of inhibition of melanogenesis by hydroquinone. Biochim. Biophys. Acta.

[7] Langfelder K., Streibel M., Jahn B., Haase G., Brakhage A.A. (2003). Biosynthesis of fungal melanins and their importance for human pathogenic fungi. Fungal. Genet. Biol..

[8] D’Ischia M., Wakamatsu K., Cicoira F., Di Mauro E., Garcia-Borron J.C., Commo S., Galván I., Ghanem G., Kenzo K., Meredith P. (2015). Melanins and melanogenesis: From pigment cells to human health and technological applications. Pigment. Cell Melanoma Res..

[9] Michalczyk-Wetula D., Wieczorek J., Płonka P.M. (2015). Splenic melanosis in agouti and black mice. Acta Biochim. Pol..

[10] Tsujii T., Seno S. (1990). Melano-macrophage centers in the aglomerular kidney of the sea horse (teleosts): Morphologic studies on its formation and possible function. Anat. Rec..

[11] Yue Y., Zhao X. (2021). Melanin-Like Nanomedicine in Photothermal Therapy Applications. Int. J. Mol. Sci..

[12] Deng R.H., Zou M.Z., Zheng D., Peng S.Y., Liu W., Bai X.F., Chen H.S., Sun Y., Zhou P.H., Zhang X.Z. (2019). Nanoparticles from Cuttlefish Ink Inhibit Tumor Growth by Synergizing Immunotherapy and Photothermal Therapy. ACS Nano.

[13] Cai J., Huang J., Ge M., Iocozzia J., Lin Z., Zhang K.Q., Lai Y. (2017). Immobilization of Pt Nanoparticles via Rapid and Reusable Electropolymerization of Dopamine on TiO_2_ Nanotube Arrays for Reversible SERS Substrates and Nonenzymatic Glucose Sensors. Small.

[14] Brzoska T., Böhm M., Lügering A., Loser K., Luger T.A. (2010). Terminal signal: Anti-inflammatory effects of α-melanocyte-stimulating hormone related peptides beyond the pharmacophore. Adv. Exp. Med. Biol..

[15] Lipton J.M., Catania A. (1998). Mechanisms of antiinflammatory action of the neuroimmunomodulatory peptide alpha-MSH. Ann. N. Y. Acad. Sci..

[16] Taylor A.W., Lee D.J. (2011). The alpha-melanocyte stimulating hormone induces conversion of effector T cells into treg cells. J. Transplant.

[17] Viljakainen L. (2015). Evolutionary genetics of insect innate immunity. Brief. Funct. Genom..

[18] Casadevall A., Rosas A.L., Nosanchuk J.D. (2000). Melanin and virulence in Cryptococcus neoformans. Curr. Opin. Microbiol..

[19] Waisberg M., Vickers B.K., Yager S.B., Lin C.K., Pierce S.K. (2012). Testing in mice the hypothesis that melanin is protective in malaria infections. PLoS ONE.

[20] Mohagheghpour N., Waleh N., Garger S.J., Dousman L., Grill L.K., Tusé D. (2000). Synthetic melanin suppresses production of proinflammatory cytokines. Cell Immunol..

[21] Plonka P.M., Passeron T., Brenner M., Tobin D.J., Shibahara S., Thomas A., Slominski A., Kadekaro A.L., Hershkovitz D., Peters E. (2009). What are melanocytes really doing all day long…?. Exp. Dermatol..

[22] Le Poole I.C., Mutis T., van den Wijngaard R.M., Westerhof W., Ottenhoff T., de Vries R.R., Das P.K. (1993). A novel, antigen-presenting function of melanocytes and its possible relationship to hypopigmentary disorders. J. Immunol..

[23] Diment S., Eidelman M., Rodriguez G.M., Orlow S.J. (1995). Lysosomal hydrolases are present in melanosomes and are elevated in melanizing cells. J. Biol. Chem..

[24] Gasque P., Jaffar-Bandjee M.C. (2015). The immunology and inflammatory responses of human melanocytes in infectious diseases. J. Infect..

[25] Hemmi H., Yoshino M., Yamazaki H., Naito M., Iyoda T., Omatsu Y., Shimoyama S., Letterio J.J., Nakabayashi T., Tagaya H. (2001). Skin antigens in the steady state are trafficked to regional lymph nodes by transforming growth factor-beta1-dependent cells. Int. Immunol..

[26] Yoshino M., Yamazaki H., Shultz L.D., Hayashi S. (2006). Constant rate of steady-state self-antigen trafficking from skin to regional lymph nodes. Int. Immunol..

[27] Park J., Brust T.F., Lee H.J., Lee S.C., Watts V.J., Yeo Y. (2014). Polydopamine-based simple and versatile surface modification of polymeric nano drug carriers. ACS Nano.

[28] Cui J., Yan Y., Such G.K., Liang K., Ochs C.J., Postma A., Caruso F. (2012). Immobilization and intracellular delivery of an anticancer drug using mussel-inspired polydopamine capsules. Biomacromolecules.

[29] Lee H., Rho J., Messersmith P.B. (2009). Facile Conjugation of Biomolecules onto Surfaces via Mussel Adhesive Protein Inspired Coatings. Adv. Mater..

[30] Tao C., Chen T., Liu H., Su S. (2019). Preparation and adsorption performance research of large-volume hollow mesoporous polydopamine microcapsules. MRS Commun..

[31] Carpentier A.F., Geinguenaud F., Tran T., Sejalon F., Martin A., Motte L., Tartour E., Banissi C. (2017). Synthetic melanin bound to subunit vaccine antigens significantly enhances CD8+ T-cell responses. PLoS ONE.

[32] Shamoto M., Osada A., Shinzato M., Kaneko C., Yoshida A. (1996). Do epidermal Langerhans cells, migrating from skin lesions, induce the paracortical hyperplasia of dermatopathic lymphadenopathy?. Pathol. Int..

[33] Wang K., Wang S., Chen K., Zhao Y., Ma X., Wang L. (2018). Doxorubicin-loaded melanin particles for enhanced chemotherapy in drug-resistant anaplastic thyroid cancer cells. J. Nanomater..

[34] Wang Z., Duan Y., Duan Y. (2018). Application of polydopamine in tumor targeted drug delivery system and its drug release behavior. J. Control Release.

[35] Zhou Z., Yan Y., Wang L., Zhang Q., Cheng Y. (2019). Melanin-like nanoparticles decorated with an autophagy-inducing peptide for efficient targeted photothermal therapy. Biomaterials.

[36] Li W.Q., Wang Z., Hao S., He H., Wan Y., Zhu C., Sun L.P., Cheng G., Zheng S.Y. (2017). Mitochondria-Targeting Polydopamine Nanoparticles to Deliver Doxorubicin for Overcoming Drug Resistance. ACS Appl Mater Interfaces.

[37] Temizoz B., Kuroda E., Ishii K.J. (2016). Vaccine adjuvants as potential cancer immunotherapeutics. Int. Immunol..

[38] Schwendener R.A. (2014). Liposomes as vaccine delivery systems: A review of the recent advances. Ther. Adv. Vaccines.

[39] Li Z., Tan S., Li S., Shen Q., Wang K. (2017). Cancer drug delivery in the nano era: An overview and perspectives (Review). Oncol. Rep..

[40] Dang Y., Guan J. (2020). Nanoparticle-based drug delivery systems for cancer therapy. Smart Mater. Med..

[41] Danhier F., Ansorena E., Silva J.M., Coco R., Le Breton A., Préat V. (2012). PLGA-based nanoparticles: An overview of biomedical applications. J. Control Release.

[42] Lee P.W., Pokorski J.K. (2018). Poly(lactic-co-glycolic acid) devices: Production and applications for sustained protein delivery. Wiley Interdiscip. Rev. Nanomed. Nanobiotechnol..

[43] Singh P., Pandit S., Mokkapati V., Garg A., Ravikumar V., Mijakovic I. (2018). Gold Nanoparticles in Diagnostics and Therapeutics for Human Cancer. Int. J. Mol. Sci..

[44] Zhang C., Zhao X., Guo H. (2018). Synergic highly effective photothermal-chemotherapy with platinum prodrug linked melanin-like nanoparticles. Artif. Cells Nanomed. Biotechnol..

[45] Mavridi-Printezi A., Guernelli M., Menichetti A., Montalti M. (2020). Bio-Applications of Multifunctional Melanin Nanoparticles: From Nanomedicine to Nanocosmetics. Nanomaterials.

[46] El-Obeid A., Al-Harbi S., Al-Jomah N., Hassib A. (2006). Herbal melanin modulates tumor necrosis factor alpha (TNF-alpha), interleukin 6 (IL−6) and vascular endothelial growth factor (VEGF) production. Phytomedicine.

[47] El-Obeid A., Alajmi H., Harbi M., Yahya W.B., Al-Eidi H., Alaujan M., Haseeb A., Trivilegio T., Alhallaj A., Alghamdi S. (2020). Distinct anti-proliferative effects of herbal melanin on human acute monocytic leukemia THP−1 cells and embryonic kidney HEK293 cells. BMC Complement. Med. Ther..

[48] Li Y., Liu X., Pan W., Li N., Tang B. (2020). Photothermal therapy-induced immunogenic cell death based on natural melanin nanoparticles against breast cancer. Chem. Commun..

[49] Araújo M., Viveiros R., Correia T.R., Correia I.J., Bonifácio V.D., Casimiro T., Aguiar-Ricardo A. (2014). Natural melanin: A potential pH-responsive drug release device. Int. J. Pharm..

[50] Park J., Moon H., Hong S. (2019). Recent advances in melanin-like nanomaterials in biomedical applications: A mini review. Biomater. Res..

[51] Liu Y.L., Ai K.L., Liu J.H., Deng M., He Y.Y., Lu L.H. (2013). Dopamine-melanin colloidal nanospheres: An efficient near-infrared photothermal therapeutic agent for in vivo cancer therapy. Adv. Mater..

[52] Yang P., Zhang S., Zhang N., Wang Y., Zhong J., Sun X., Qi Y., Chen X., Li Z., Li Y. (2019). Tailoring Synthetic Melanin Nanoparticles for Enhanced Photothermal Therapy. ACS Appl. Mater. Interfaces.

[53] Jiang Q., Liu Y., Guo R., Yao X., Sung S., Pang Z., Yang W. (2019). Erythrocyte-cancer hybrid membrane-camouflaged melanin nanoparticles for enhancing photothermal therapy efficacy in tumors. Biomaterials.

[54] Wang X., Zhang J., Wang Y., Wang C., Xiao J., Zhang Q., Cheng Y. (2016). Multi-responsive photothermal-chemotherapy with drug-loaded melanin-like nanoparticles for synergetic tumor ablation. Biomaterials.

[55] Wang W., Jing T., Xia X., Tang L., Huang Z., Liu F., Wang Z., Ran H., Li M., Xia J. (2019). Melanin-loaded biocompatible photosensitive nanoparticles for controlled drug release in combined photothermal-chemotherapy guided by photoacoustic/ultrasound dual-modality imaging. Biomater. Sci..

[56] Manolova V., Flace A., Bauer M., Schwarz K., Saudan P., Bachmann M.F. (2008). Nanoparticles target distinct dendritic cell populations according to their size. Eur. J. Immunol..

[57] Cuzzubbo S., Banissi C., Rouchon M.S., Tran T., Tanchot C., Tartour E., Carpentier A.F. (2020). The adjuvant effect of melanin is superior to incomplete Freund’s adjuvant in subunit/peptide vaccines in mice. Cancer Immunol. Immunother..

[58] Wang N., Yang Y., Wang X., Tian X., Qin W., Wang X., Liang J., Zhang H., Leng X. (2019). Polydopamine as the Antigen Delivery Nanocarrier for Enhanced Immune Response in Tumor Immunotherapy. ACS Biomater. Sci. Eng..

[59] Nieto C., Vega M.A., Marcelo G., Del Valle E.M.M. (2018). Polydopamine nanoparticles kill cancer cells. RSC Adv..

[60] Oberländer U., Pletinckx K., Döhler A., Müller N., Lutz M.B., Arzberger T., Riederer P., Gerlach M., Koutsilieri E., Scheller C. (2011). Neuromelanin is an immune stimulator for dendritic cells in vitro. BMC Neurosci..

[61] Zecca L., Wilms H., Geick S., Claasen J.H., Brandenburg L.O., Holzknecht C., Panizza M.L., Zucca F.A., Deuschl G., Sievers J. (2008). Human neuromelanin induces neuroinflammation and neurodegeneration in the rat substantia nigra: Implications for Parkinson’s disease. Acta Neuropathol..

[62] Tajima K., Yamanaka D., Ishibashi K.I., Adachi Y., Ohno N. (2019). Solubilized melanin suppresses macrophage function. FEBS Open Bio..

[63] Chen Q., Xu L., Liang C., Wang C., Peng R., Liu Z. (2016). Photothermal therapy with immune-adjuvant nanoparticles together with checkpoint blockade for effective cancer immunotherapy. Nat. Commun..

[64] Chen W., Qin M., Chen X., Wang Q., Zhang Z., Sun X. (2018). Combining photothermal therapy and immunotherapy against melanoma by polydopamine-coated Al2O3 nanoparticles. Theranostics.

[65] Rong L., Zhang Y., Li W.S., Su Z., Fadhil J.I., Zhang C. (2019). Iron chelated melanin-like nanoparticles for tumor-associated macrophage repolarization and cancer therapy. Biomaterials.

[66] Ye Y., Wang C., Zhang X., Hu Q., Zhang Y., Liu Q., Wen D., Milligan J., Bellotti A., Huang L. (2017). A melanin-mediated cancer immunotherapy patch. Sci. Immunol..

[67] Zhu X., Feng W., Chang J., Tan Y.W., Li J., Chen M., Sun Y., Li F. (2016). Temperature-feedback upconversion nanocomposite for accurate photothermal therapy at facile temperature. Nat. Commun..

[68] He Y., Cong C., Li X., Zhu R., Li A., Zhao S., Li X., Cheng X., Yang M., Gao D. (2019). Nano-drug System Based on Hierarchical Drug Release for Deep Localized/Systematic Cascade Tumor Therapy Stimulating Antitumor Immune Responses. Theranostics.

